# Analysis of Online Resource Utilization Among Individuals With Vitiligo

**DOI:** 10.7759/cureus.100606

**Published:** 2026-01-02

**Authors:** Nneka Ede, Prachi Khanna, Beth Schneider, Ammar Ahmed

**Affiliations:** 1 Dermatology, Dell Medical School at The University of Texas at Austin, Austin, USA; 2 Informatics, MyHealthTeam, San Francisco, USA

**Keywords:** internet, online health resources, online resources, quality of life, vitiligo

## Abstract

Background

Few studies have assessed the usage of online resources by vitiligo patients. Our goal was to determine what information and sources individuals with vitiligo seek out on the internet and how this information affects them.

Methods

A 15-question cross-sectional survey related to online resources and their impact was developed and distributed to users on the MyVitiligoTeam forum from May to June 2022. The questionnaire gathered information on participants’ demographics, their engagement with online resources, and the impact of this information. Descriptive statistics were completed with Qualtrics (Provo, Utah, United States).

Results

There were 95 responses. The majority of respondents (n = 72, 79.12%) reported currently using online resources to talk about or learn about vitiligo, with the most frequently utilized resources being medical websites (n = 35, 55.56%), vitiligo-specific organizations (n = 30, 47.62%), and social media (n = 21, 33.33%). When asked about the most useful resources for managing vitiligo, participants prioritized vitiligo-specific websites (37.25%) and social media (17.65%). The most important reasons for seeking out information online were for seeking out medical advice from healthcare professionals or organizations, learning about the cause of vitiligo, and exploring treatment experiences. The biggest impact yielded by online resources has been learning to cope with and manage psychosocial aspects of the condition (n = 20, 37.74%).

Conclusions

This study reveals the depth of influence that online resources have on vitiligo patients as they cope and manage their disease, particularly the significant psychosocial benefits. The findings show the psychosocial benefits of online engagement in supporting vitiligo patients.

## Introduction

Vitiligo is a condition that causes irregular patches of complete depigmentation due to destruction of melanocytes; its prevalence is ~0.5-2% worldwide [1]. The content and accuracy of dermatology-related information on the internet have been studied extensively, in general, and in the context of specific dermatologic conditions [2,3]. With regard to vitiligo, studies have analyzed the accuracy of content on social media sites (i.e., YouTube and Twitter) [4,5] and trends in Google searches for vitiligo [6]. However, the views of individuals with vitiligo about these online resources and their utility have not been extensively studied.

This study aims to explore the utilization of online resources by individuals affected by vitiligo. The main objectives of this study are to identify the demographics of users living with vitiligo who are active on the internet, determine the information they explore online, and assess how said resources lead to lifestyle changes and impact disease management.

The findings in this research were previously presented as a meeting abstract at the Global Vitiligo Foundation Annual Scientific Symposium on March 16, 2023.

## Materials and methods

Study design and participants

The study employs a cross-sectional survey design to assess online resource utilization in individuals with vitiligo. It was distributed to users of MyVitiligoTeam, a San Francisco-based social network and online support group with over 11,000 members. The sample consisted of 95 individuals. The study was conducted entirely online over a two-month period. Data were collected from participants located wherever they accessed the MyVitiligoTeam platform.

The exact sampling criteria for which eligible active users received the survey were determined by the MyVitiligoTeam platform’s outreach protocols. Eligibility criteria included speaking English, being over the age of 18, having a self-reported diagnosis of vitiligo, and being an active forum user. Specifically, active users were those who engaged with the platform either by opening an email or visiting the website within at least a year of the collection period. Individuals under 18 and those without a reported diagnosis of vitiligo were excluded.

Study instrument and data collection

Survey collection was completed with a structured online questionnaire developed by authors PK and AA, based on a review of existing literature on online health information seeking behavior and drawing upon a 2020 survey studying hidradenitis suppurativa patients [7]. No pilot testing was done; questions underwent internal review by PK and AA to ensure clarity, relevance to study objectives, and appropriateness for the target population. Based on this, questions were refined to ensure face validity and content validity.

The 15-question survey gathered information on participants’ demographics, their engagement with online resources related to vitiligo, the types of information they sought, the sources they utilized, and the perceived impact of this information on their disease management and well-being. It included a mix of multiple-choice, Likert scale [8], and open-ended questions and can be found in its entirety in Appendix A. Participants were not required to answer every question. The survey was conducted online, administered via MyVitiligoTeam, and live on the platform from May to June 2022.

Statistical analysis

No formal a priori sample size calculation was performed. Descriptive statistics, including means, percentages, frequencies, and SDs, were completed with Qualtrics (Provo, Utah, United States). Stats were used to summarize participants’ demographics and detail their reported online resource usage behaviors and the impact of said resources. Multiple-choice responses were tallied to determine frequencies and percentages. Open-ended responses were thematically analyzed by grouping similar responses into categories before frequencies and percentages were calculated. Likert scale questions were rated on a 5-point scale, ranging from 1 (Not at all important) to 5 (Extremely important). Specifically, within the questionnaire, respondents were asked to rank the importance of ten reasons why they use online tools. The numerical responses provided by participants to each reason were averaged together and reported as the mean importance score (M). For many questions, multiple responses were permitted, allowing the sum of responses to exceed the total number of participants (N).

Ethical considerations

The University of Texas at Austin Institutional Review Board (IRB) determined that this protocol met the criteria for exemption from IRB review (STUDY00002314). Personal identifiers were not recorded, and all data were securely stored.

## Results

There were 95 total responses from participants who were primarily White, not Hispanic or Latino (n = 39, 52%), and predominantly female (n = 74, 77.89%), with a mean age of 60 years (SD = 12.5 years). The majority of respondents (n = 90, 94.74%) have received an official vitiligo diagnosis from a healthcare provider (Table 1).

**Table 1:** Demographic characteristics

Survey question	Response	n	%
First, what is your age? (N = 95)	Average age (years)	60	
Which of the following best describes you? (N = 95)	Male	21	22.11%
	Female	74	77.89%
	Non-binary	0	0%
	Prefer not to say	0	0%
Have you received an official vitiligo diagnosis from a healthcare provider? (N = 95)	Yes	90	94.74%
	No, and I don’t plan to see a doctor to get a diagnosis	4	4.21%
	No, but I plan to see a doctor to get a diagnosis	1	1.05%
Finally, which one of the following best describes your ethnicity? (N = 75)	White	39	52%
	Black or African American	16	21.33%
	Hispanic or Latino	14	18.67%
	American Indian or Alaskan native	2	2.67%
	Native Hawaiian or Other Pacific Islander	2	2.67%
	Prefer not to say	2	2.67%

Usage of online sources

Respondents reported using a variety of online sources to understand their vitiligo symptoms. When asked which resources they currently used, general medical websites were ranked the most frequently used resource (n = 35, 55.56%), followed by vitiligo-specific organizations (n = 30, 47.62%), social media (n = 21, 33.33%), online video platforms (n = 16, 25.4%), and forums (n = 12, 19.05%). Only one respondent (n = 1, 1.59%) indicated currently using other sources (Table 2).

**Table 2:** Resources utilized by individuals with vitiligo

Survey question	Response	n	%
Have you looked for information about vitiligo from any of the following online sources to help you understand your symptoms? (N = 95) (multiple selection)	Vitiligo-specific organizations	95	100%
	Online video platform (YouTube, etc.)	25	26.32%
	Social media (Facebook, Instagram, TikTok, Twitter, etc.)	28	29.47%
	Forums/blog posts: (Reddit, etc.)	20	21.05%
	Other	6	6.32%
	Medical websites (WebMD, Healthline, etc.)	65	68.42%
Do you currently use online resources to learn and/or talk about vitiligo? (N = 91)	Yes	72	79.12%
	No	19	20.88%
What sources do you currently use? (N = 63) (multiple selection)	Medical websites	35	55.56%
	Vitiligo-specific organizations	30	47.62%
	Social media	21	33.33%
	Online video platform	16	25.40%
	Forums/blog posts	12	19.05%
	Other	1	1.59%
What online source of information has been most useful for managing and living with vitiligo? (N = 51) (open-ended)	Vitiligo-specific websites	19	37.25%
	Social media	9	17.65%
	General medical websites	5	9.80%
	None	10	19.61%
	Others	8	15.69%

Impact of online sources

Participants were asked what has been important to them when using online tools to help manage their vitiligo, scoring 10 reasons for seeking out information online from Not at all important (1) to Extremely important (5) (Table 3). Seeking out medical advice about vitiligo from a healthcare provider or group (M = 4.21, SD = 1.07) and learning about the causes of vitiligo (M = 4.19, SD = 1.26) were deemed most important. Other highly rated aspects included seeking treatment experiences from other patients (M = 4.15, SD = 1.15), learning about prescriptions (M = 4.12, SD = 1.26), receiving support and advice on coping (M = 4.08, SD = 1.27), finding doctors who treat vitiligo (M = 4.05, SD = 1.22), exploring over-the-counter treatments (M = 4.03, SD = 1.26), and lifestyle changes (M = 3.97, SD = 1.33).

**Table 3:** Importance of reasons for using online resources rated on a 5-point Likert scale Mean importance score (M) = average score on a scale from 1 to 5

What has been important to you when using online tools to help you manage vitiligo? (N = 75)	Mean importance score (M)	Importance ranking	n	%
Seeking out medical advice about vitiligo from a healthcare provider, group, or foundation	4.21	1 - Not at all important	3	4%
		5 - Extremely important	39	52%
Learning about the cause of my vitiligo	4.19	1 - Not at all important	6	8%
		5 - Extremely important	46	61.33%
Seeking out treatment experiences from other patients living with vitiligo	4.15	1 - Not at all important	5	6.67%
		5 - Extremely important	38	50.67%
Learning about prescriptions and other doctor-prescribed treatments	4.12	1 - Not at all important	6	8%
		5 - Extremely important	42	56%
Support and advice on coping with vitiligo	4.08	1 - Not at all important	8	10.67%
		5 - Extremely important	39	52%
Finding a doctor who treats vitiligo	4.05	1 - Not at all important	5	6.67%
		5 - Extremely important	38	50.67%
Exploring over-the-counter treatments for vitiligo	4.03	1 - Not at all important	8	10.67%
		5 - Extremely important	36	48%
Lifestyle changes to help with my vitiligo	3.97	1 - Not at all important	8	10.67%
		5 - Extremely important	37	49.33%
Finding out why I was getting patches/spots on my skin	3.79	1 - Not at all important	11	14.67%
		5 - Extremely important	37	49.33%
Learning about camouflage options	3.77	1 - Not at all important	11	14.67%
		5 - Extremely important	31	41.33%

Participants were also asked to describe the impact of these resources on managing and living with vitiligo in an open-ended question. The top responses centered around coping and knowledge (n = 20, 37.74%, and n = 13, 24.53%, respectively). Participants specifically noted “Seeing others with the same condition has helped me understand I am not alone,” “Knowing there is a large community,” and “Hope for new treatments and new melanocyte transplant techniques.” However, over a quarter (n = 14, 26.42%) of individuals with vitiligo felt online sources were not helpful at all. One respondent noted, “Nothing has helped me to live with it. I think it is something you have to come to accept on your own.”

A majority of respondents (n = 47, 62.67%) did not share their experiences or advice online, whereas 28 (37.33%) respondents did engage in sharing. For those who shared advice online, coping strategies were the most common type of advice given (n = 12, 57.14%), followed by treatment and camouflage advice (n = 7, 33.33%) and other types of advice (n = 2, 9.52%), as seen in Table 4.

**Table 4:** Information shared and sought after online among individuals with vitiligo

Survey question	Response	n	%
In what ways, if any, have online sources of information helped you manage and live with vitiligo? (N = 53) (open-ended)	Coping	20	37.74%
	Not helpful	14	26.42%
	Knowledge	13	24.53%
	Learning about new treatments	4	7.55%
	Other	2	3.77%
Do you feel that the information you found online helped you feel supported in your journey with vitiligo? (N = 75)	Yes	56	74.67%
	No	19	25.33%
Have you given advice or shared your experiences with other vitiligo patients online? (N = 75)	No	47	62.67%
	Yes	28	37.33%
What advice have you given to others online? (N = 21) (open-ended)	Coping advice	12	57.14%
	Treatment and camouflage	7	33.33%
	Other	2	9.52%

Impact on diagnosis and treatment decisions

When asked about the usefulness of information found online in aiding their diagnosis, the majority (n = 60, 69.77%) of respondents reported that the information did not help them get an easier or faster diagnosis from their doctor, while a few (n = 26, 30.23%) felt that it did. However, information found online did influence treatment decisions for many respondents. As a result of exploring information online, almost 75% (n = 56) of participants changed their treatment decisions and either talked to their doctor about specific treatments, sought over-the-counter treatment options, applied for clinical trials, or decided against a form of treatment. Specifically, 37.33% (n = 28) decided to talk to their doctor about treatment options, while 25.33% (n = 19) reported that the information did not affect their treatment decisions. Sixteen (21.33%) respondents decided not to pursue any treatment, and another 16 (21.33%) respondents opted for over-the-counter treatments. About 10% (n = 8) applied for clinical trials based on information they found online (Table 5).

**Table 5:** Impact of online resources on vitiligo diagnosis and treatment decisions

Survey question	Response	n	%
Do you feel that the information you found from other sources made it easier for you to get a diagnosis of vitiligo from your healthcare provider? (N = 86)	No, the information did not help me get a diagnosis easier/faster from my doctor	60	69.77%
	Yes	26	30.23%
In what ways has the information you read affected your treatment decisions for your vitiligo? (N = 75) (Multiple Selection)	I decided to talk to my doctor about treatment	28	37.33%
	It did not affect my treatment decision	19	25.33%
	I decided not to take any treatment	16	21.33%
	I decided to take some form of over-the-counter treatment	16	21.33%
	I decided to apply for a clinical trial	8	10.67%

## Discussion

In this study, the most frequently accessed online sources patients used to learn more about their vitiligo were both general medical websites and vitiligo-specific websites, followed by social media. In a 2025 study looking at internet resources in the broader context of outpatient dermatology, results showed that dermatologic and general medical websites were most preferred when searching for dermatology information [9]. Our results validate this finding and specifically reveal this same trend to be true for vitiligo patients. However, when respondents were asked what they deemed to be the most helpful internet resource, a majority specified vitiligo-specific websites and social media; very few noted general medical websites. This suggests that patients may gravitate towards vitiligo-specific websites and social media for more sustained engagement, likely due to the content within these sources. These sources tend to have more body-positive content, which a 2022 study found to be the most important reason for internet engagement for vitiligo patients [10].

This study’s findings also show that among patients with vitiligo who are active on the internet, online resources can affect the ways they approach their disease; however, the effectiveness and impact of resources vary. The majority of participants used resources to deepen their knowledge about their condition and explore medical treatment options. This aligns with a previous study [11] that revealed the top two reasons its participants used the internet were to help understand their dermatologic diagnosis and seek therapy. Along that same line, our survey showed that participants ranked seeking medical advice and treatment options as highly important reasons for using the internet for their vitiligo. Online information significantly influenced respondents’ treatment decisions, with many opting to discuss treatment options with their doctors, changing their treatment decisions, or enrolling in clinical trials. A 2022 cross-sectional study looking at internet usage in psoriasis patients had similar findings, with patients reporting searching for medication and treatment options as the second most common use of social media for their condition [12]. These results allude to the fact that engagement with online resources and social media may indirectly allow for more robust dialogue between patients and providers about treatment.

Many studies have emphasized the importance of prioritizing psychosocial health in the management of vitiligo [13-15]. A 2022 qualitative study looking at the views of vitiligo patients using online forums found that patients use the internet as a way to tell their story, engage with others with similar stories, and provide advice [16]. Our study supports this finding but also elaborates on how online resources make users feel, revealing that 56 (74.67%) respondents found the internet helpful in making them feel more supported on their vitiligo journey. These findings echo results of similar studies that look at disease-specific online peer support groups in alopecia and rosacea patients, which show that these types of groups provide emotional support and an avenue to connect with others with shared experiences [17,18].

While a vast majority of respondents have sought an official diagnosis, the role of online information in expediting this process appears limited based on our results, as a majority of respondents (n = 60, 69.77%) did not find online information helpful in receiving a diagnosis faster or easier. This suggests that while online resources are valuable for ongoing support and education, they may have limited impact on hastening diagnosis. This result is congruent with the finding that individuals with vitiligo frequently feel frustrated with the help-seeking process both online and from providers [19].

This study can aid providers by making them aware of the significant influence of online resources on patients’ well-being, how these resources affect patients’ knowledge of their disease processes and treatment options, and the limitations of these resources. These findings can help foster active discussions about online resources during clinic appointments and help providers set expectations for their patients about the information they find online. Future research should focus on further cross-sectional analyses with larger sample sizes across multiple social media bases to enhance generalizability and longitudinal analyses to study the long-term effects of social media on vitiligo patients. Research should also focus on inferential statistical analysis to establish more causal relationships.

Limitations to this study are related to its lack of generalizability due to a single target population, lack of a priori sample size calculation, and relatively small sample size. While the study design allowed us to engage with individuals actively seeking information online, our sample may not be representative of the broader vitiligo community and may result in selection bias. Results may differ from patients not on this particular social network or active on the Internet in general. No pilot testing of the questionnaire was conducted, meaning possible ambiguities in questions may have impacted participants’ responses.

## Conclusions

Our survey shows that online resources can be a source of support and can impact the decisions patients make regarding their vitiligo and how they cope with their disease. The results from this study are important for providers who treat patients with vitiligo to both understand the online ecosystem and help manage patient expectations regarding the utility of online resources. These data may help inform the creation of new online content to fill gaps regarding information that individuals with vitiligo seek.

## Appendices

Appendix A 

**Table 6:** Survey questionnaire instrument

Survey question		Response option
First, what is your age?		Open-ended
Which of the following best describes you?		Male
		Female
		Non-binary
		Prefer not to say
Have you received an official vitiligo diagnosis from a healthcare provider?		Yes
		No, and I don’t plan to see a doctor to get a diagnosis
		No, but I plan to see a doctor to get a diagnosis
Have you looked for information about vitiligo from any of the following online sources to help you understand your symptoms?		Vitiligo-specific organizations
		Online video platform (YouTube, etc.)
		Social media (Facebook, Instagram, TikTok, Twitter, etc.)
		Forums/blog posts: (Reddit, etc.)
		Other (internet, medical journals, doctor, Loma Linda Hospital, and books)
		Medical websites (WebMD, Healthline, etc.)
Do you feel that the information you found from other sources made it easier for you to get a diagnosis of vitiligo from your healthcare provider?		Yes/No
Do you currently use online resources to learn and/or talk about vitiligo?		Yes/No
What sources do you currently use? Please check all that apply.		Medical websites
		Vitiligo-specific organizations
		Social media
		Online video platform
		Forums/blog posts
		Other
What has been important to you when using online tools to help you manage vitiligo?	Seeking out medical advice about vitiligo from a healthcare provider, group, or foundation	Likert scale (1-5)
	Learning about the cause of my vitiligo	Likert scale (1-5)
	Seeking out treatment experiences from other patients living with vitiligo	Likert scale (1-5)
	Learning about prescriptions and other doctor-prescribed treatments	Likert scale (1-5)
	Support and advice on coping with vitiligo	Likert scale (1-5)
	Finding a doctor who treats vitiligo	Likert scale (1-5)
	Exploring over-the-counter treatments for vitiligo	Likert scale (1-5)
	Lifestyle changes to help with my vitiligo	Likert scale (1-5)
	Finding out why I was getting patches/spots on my skin	Likert scale (1-5)
	Learning about camouflage options	Likert scale (1-5)
What online source of information has been most useful for managing and living with vitiligo?		Vitiligo-specific websites
		Social media
		General medical websites
		None
		Others
In what ways, if any, have online sources of information helped you manage and live with vitiligo?		Open-ended
In what ways has the information you read affected your treatment decisions for your vitiligo?		Open-ended
Do you feel that the information you found online helped you feel supported in your journey with vitiligo?		Yes/No
Have you given advice or shared your experiences with other vitiligo patients online?		Yes/No
What advice have you given to others online?		Open-ended
Finally, which one of the following best describes your ethnicity?		White
		Black or African American
		Hispanic or Latino
		American Indian or Alaskan native
		Native Hawaiian or Other Pacific Islander
		Prefer not to say
